# Supplementary material for: Semi-synthetic cinnamodial analogues: Structural insights into the insecticidal and antifeedant activities of drimane sesquiterpenes against the mosquito Aedes aegypti
Source: PLoS Negl Trop Dis. 2020 Feb 26;14(2):e0008073. doi: 10.1371/journal.pntd.0008073 (PMC7062286; doi:10.1371/journal.pntd.0008073)
Supplement: S1 File — Supporting structures, larvicidal concentration-response curve, synthesis, characterization data (including 1H and 13C NMR, IR, and mass spectra), and supporting references. (DOCX) [file pntd.0008073.s001.docx]

Supporting Information

**Semi-synthetic cinnamodial analogues: structural insights into the insecticidal and antifeedant activities of drimane sesquiterpenes against the mosquito *Aedes aegypti***

*Preston K. Manwill^1,3^, Megha Kalsi^2^, Sijin Wu^1^, Erick M. Rodriguez^2^, Xiaolin Cheng^1^, Peter M. Piermarini^2,3^, and Harinantenaina L. Rakotondraibe^1,3^*

*Division of Medicinal Chemistry and Pharmacognosy, College of Pharmacy, The Ohio State University^1^; Department of Entomology, The Ohio State University, Ohio Agricultural Research and Development Center, Wooster, Ohio, United States of America^2^; Center for Applied Plant Sciences, The Ohio State University^3^*

Table of Contents

[Supporting Structures 4](#_Toc24012269)

[Larvicidal Concentration-Response Curve 5](#_Toc24012270)

[Synthesis 5](#_Toc24012272)

[Cinnamodial (1), 9](#_Toc24012273)

[Cinnamodiacid (5), 10](#_Toc24012274)

[Cinnamodimethylester (6), 13](#_Toc24012275)

[Cinnamo-N,11-dihydro-11-pyridazinol (7), 16](#_Toc24012276)

[7*ß*-hydroxy-cinnamopyridazine (18), 19](#_Toc24012277)

[6-*O*-acetyl-12*α*-methyl-pereniporin A (8), 21](#_Toc24012278)

[6-*O*-acetyl-12a-methyl-pereniporin A (9), 24](#_Toc24012279)

[(-)-6*ß*-Acetoxy-9*α*-hydroxydrim-7-ene-12-methyl-12-one-11-al (10), 27](#_Toc24012280)

[Cinnamodial 12-ethylene acetal (11), 29](#_Toc24012281)

[12*α*/*ß*-methyl-pereniporin A (12), 31](#_Toc24012282)

[(1’*S*/*R*)-1’-((8a*S*)-5,5,8a-trimethyl-1,4-dioxo-1,4,4a,5,6,7,8,8a-octahydronaphthalene-2-yl)ethyl formate (13), 34](#_Toc24012283)

[Cinnamothiazolidine (16), 37](#_Toc24012284)

[References 39](#_Toc24012285)

Table of Figures

[**Figure S1**. Structure of CDIAL and semi-synthetic derivatives. 4](#_Toc24012575)

[**Figure S2**. Structure of cytotoxic quinones. 4](#_Toc24012576)

[**Figure S3**. Structure of reactive *α*,*ß*-unsaturated aldehydes and an isothiocyanate. 5](#_Toc24012577)

[**Figure S4.** Concentration-response curves for larvicidal efficacies of CDIAL (**1**) and **13**. 5](#_Toc24012578)

[**Figure S5**. Proton NMR spectra (top) and carbon NMR spectra (bottom) of compound **1** in CDCl_3_ at 400 and 100 MHz, calibrated to Chloroform-*d* 7.26 (1) and 77.36 (3). 9](#_Toc24012579)

[**Figure S6**. Proton NMR spectra (Full and inset) of compound **5** in CD_3_OD at 400 MHz, calibrated to Methanol-*d*_4_ 3.34 (5). 10](#_Toc24012580)

[**Figure S7**. Carbon NMR spectra of compound **5** in CD_3_OD at 100 MHz, calibrated to Methanol*-d*_4_ 49.86 (7). 11](#_Toc24012581)

[**Figure S8**. IR spectrum of compound **5**. 12](#_Toc24012582)

[**Figure S9**. HRESIMS of compound **5**. 12](#_Toc24012583)

[**Figure S10**. Proton NMR spectra (Full and inset) of compound **6** in CDCl_3_ at 400 MHz, calibrated to Chloroform*-d* 7.26 (1). 13](#_Toc24012584)

[**Figure S11**. Carbon NMR spectra of compound **6** in CDCl_3_ at 100 MHz, calibrated to Chloroform*-d* 77.36 (3). 14](#_Toc24012585)

[**Figure S12**. IR spectrum of compound **6**. 15](#_Toc24012586)

[**Figure S13**. HRESIMS of compound **6**. 15](#_Toc24012587)

[**Figure S14**. Proton NMR spectra of compound **7** in CD_3_CN (top), calibrated to acetonitrile*-d*_3_ 1.96 (5), and CD_3_OD (bottom), calibrated to Methanol*-d*_4_ 3.34 (5), at 400 MHz. 16](#_Toc24012588)

[**Figure S15**. Carbon NMR spectra of compound **7** in CD_3_CN at 100 MHz, calibrated to acetonitrile*-d*_3_ 118.77 (1). 17](#_Toc24012589)

[**Figure S16**. IR spectrum of compound **7**. 18](#_Toc24012590)

[**Figure S17**. HRESIMS of compound **7**. 18](#_Toc24012591)

[**Figure S18**. Proton NMR spectra (top) and carbon NMR spectra (bottom) of compound **18** in CD_3_OD at 400 and 100 MHz, calibrated to calibrated to Methanol*-d*_4_ 3.34 (5) and 49.86 (7), respectively. 19](#_Toc24012592)

[**Figure S19**. IR spectrum of compound **18**. 20](#_Toc24012593)

[**Figure S20**. Proton NMR spectra (Full and inset) of compound **8** in CDCl_3_ at 400 MHz, calibrated to Chloroform*-d* 7.26 (1). 21](#_Toc24012594)

[**Figure S21**. Carbon NMR spectra of compound **8** in CDCl_3_ at 100 MHz, calibrated to Chloroform*-d* 77.36 (3). 22](#_Toc24012595)

[**Figure S22**. Proton NMR spectra (Full and inset) of compound **8** in CDCl_3_ at 400 MHz, calibrated to Chloroform*-d* 7.26 (1). Proton taken after 46 day incubation in deuterated chloroform. 22](#_Toc24012596)

[**Figure S23**. IR spectrum of compound **8**. 23](#_Toc24012597)

[**Figure S24**. HRESIMS of compound **8**. 23](#_Toc24012598)

[**Figure S25**. Proton NMR spectra (Full and inset) of compound **9** in CDCl_3_ at 400 MHz, calibrated to Chloroform*-d* 7.26 (1). 24](#_Toc24012599)

[**Figure S26**. Carbon NMR spectra of compound **9** in CDCl_3_ at 100 MHz, calibrated to Chloroform*-d* 77.36 (3). 25](#_Toc24012600)

[**Figure S27**. IR spectrum of compound **9**. 26](#_Toc24012601)

[**Figure S28**. HRESIMS of compound **9**. 26](#_Toc24012602)

[**Figure S29**. Proton NMR spectra (top) and carbon NMR spectra (bottom) of compound **10** in CDCl_3_ at 400 and 100 MHz, calibrated to Chloroform*-d* 7.26 (1)and 77.36 (3), respectively. 27](#_Toc24012603)

[**Figure S30**. IR spectrum of compound **10**. 28](#_Toc24012604)

[**Figure S31**. HRESIMS of compound **10**. 28](#_Toc24012605)

[**Figure S32***.* Proton NMR spectra (top) and carbon NMR spectra (bottom) of compound **11** in CDCl_3_ at 400 MHz and 100 MHz, calibrated to Chloroform*-d* 7.26 (1)and 77.36 (3), respectively. 29](#_Toc24012606)

[**Figure S33**. IR spectrum of compound **11**. 30](#_Toc24012607)

[**Figure S34**. HRESIMS of compound **11**. 30](#_Toc24012608)

[**Figure S35**. NMR spectra (Full and inset) of compound **12** in CDCl_3_ at 400 MHz, calibrated to Chloroform*-d* 7.26 (1). 31](#_Toc24012609)

[**Figure S36**. Carbon NMR spectra (full and inset) of compound **12** in CDCl_3_ at 100 MHz, calibrated to Chloroform*-d* 77.36 (3). 32](#_Toc24012610)

[**Figure S37**. IR spectrum of compound **12**. 33](#_Toc24012611)

[**Figure S38**. HRESIMS of compound **12**. 33](#_Toc24012612)

[**Figure S39**. NMR spectra (Full and inset) of compound **13** in CDCl_3_ at 400 MHz, calibrated to Chloroform*-d* 7.26 (1). 34](#_Toc24012613)

[**Figure S40**. Carbon NMR spectra (full and inset) of compound **13** in CDCl_3_ at 100 MHz, calibrated to Chloroform*-d* 77.36 (3). 35](#_Toc24012614)

[**Figure S41**. IR spectrum of compound **13**. 36](#_Toc24012615)

[**Figure S42**. HRESIMS of compound **13**. 36](#_Toc24012616)

[**Figure S43**. Proton NMR spectra (top) and carbon NMR spectra (bottom) of compound **16** in Pyridine at 400 MHz and 100 MHz, calibrated to Pyridine*-d*_5_ 7.22 (1) and 123.87 (3), respectively. 37](#_Toc24012617)

[**Figure S44** IR spectrum of compound **16**. 38](#_Toc24012618)

[**Figure S45**. HRESIMS of compound **16**. 38](#_Toc24012619)

# Supporting Structures

**Figure S1**. Structure of CDIAL and semi-synthetic derivatives.

**Figure S2**. Structure of cytotoxic quinones.

**Figure S3**. Structure of reactive α,ß-unsaturated aldehydes and an isothiocyanate.

# Larvicidal Concentration-Response Curve

#

**Figure S4.** Concentration-response curves for larvicidal efficacies of CDIAL (**1**) and **13**. The efficacy values were calculated using Abbott’s correction to account for control (100% acetone) mortality. Values are means ± SEM based on 8 replicates of 5 larvae per concentration. Lines were fitted to the data and best fit EC_50_ values were determined in GraphPad Prism 6.0 using the ‘log(agonist) vs. normalized response -- Variable slope’ function. See text for EC_50_ values.

# Synthesis

**Cinnamodiacid (5):** Sodium phosphate monobasic monohydrate (11 mg, 0.08 mmol) in 0.2 mL of water and H_2_O_2_ (66 μL, 0.64 mmol) were added to a solution of **1** (50.0 mg, 0.162 mmol) dissolved in 2 mL of acetonitrile. Sodium chlorite (73.5 mg, 0.8 mmol) in 0.5 mL of water was added dropwise over a period of 15 min. The reaction mixture was stirred for 24 h at room temperature. Solid sodium sulfite, Na_2_SO_3_ (5 mg) was added and the mixture was stirred for 5 min then acidified by an aqueous solution of 10% HCl. Aqueous layer was washed three times with CH_2_Cl_2_. Combined organic layers were washed with brine, dried over MgSO_4_, filtered, and concentrated *in vacuo*. Crystallization in chloroform afforded diacid **5** (43.98 mg, 81%) as colorless crystals; ${[\alpha]}_{D}^{20}$ −188 (*c* 1, ACN); UV (ACN) λ_max_ (log ε 3.69) 204.5 nm; IR (KBr) v_max_ 3435, 3172, 2951, 2929, 2871, 1738, 1395, 1371, 1236, 1073, 1031, 957 and 756 cm^−1^; ^1^H NMR (400 MHz, MeOH) δ 6.86 (1H, d, *J* = 5.1 Hz, H-7), 5.74 (1H, t, *J* = 4.8 Hz, H-6), 2.12 (3H, s, H-17), 2.10 (1H, d, *J* = 4.7 Hz, H-5), 2.09 (1H, td, *J* = 13.7, 3.8 Hz, H-1α), 1.72 (1H, qt, *J* = 13.5, 3.1 Hz, H-2ß), 1.55 (1H, dquint, *J* = 13.7, 3.4 Hz, H-2α), 1.43 (1H, dq, *J* = 12.8, 2.7 Hz, H-3ß), 1.32 (1H, td, *J* = 13.1, 3.1 Hz, H-3α), 1.27 (1H, dm, *J* = 13.8 Hz, H-1ß), 1.29 (3H, s, H-15), 1.22 (3H, s, H-14), 1.04 (3H, s, H-13); ^13^C NMR (100 MHz, MeOD) δ 177.1 (C, C-11), 172.8 (C, C-16), 170.3 (C, C-12), 137.2 (CH, C-7), 136.8 (C, C-8), 79.6 (C, C-9), 68.1 (CH, C-6), 46.3 (CH_2_, C-3), 46.1 (CH, C-5), 42.7 (C, C-10), 35.5 (C, C-4), 34.5 (CH_2_, C-1), 34.2 (CH_3_, C-13), 26.0 (CH_3_, C-14), 22.3 (CH_3_, C-17), 21.2 (CH_3_, C-15), 20.1 (C, C-2); HRESIMS *m/z* 363.14104 [M + Na]^+^ (calcd for C_17_H_24_O_7_Na, 363.14147).

**Cinnamodimethylester (6):** To a solution of **5** (50.6 mg, 0.149 mmol) in 1 mL methanol–diethyl ether (1:1), (Trimethylsilyl)diazomethane (80.0 mg, 0.700 mmol, 2.0 M) in hexanes (0.350 mL) was added dropwise at 0 ^o^C. The reaction mixture was stirred at 0 ^o^C for 15 min, concentrated *in vacuo* and purified via column chromatography (hexanes–EtOAc 3:1) to yield dimethyl ester **6** (39.41 mg, 72%) as a colorless oil: ${[\alpha]}_{D}^{20}$ −161 (*c* 1, ACN); IR (KBr) ν_max_ 3501, 2949, 2927, 2868, 1738, 1456, 1371, 1233, 1077, 1033, 754, and 700 cm^−1^; ^1^H NMR (400 MHz, CDCl_3_) δ 6.98 (1H, d, *J* = 5.4 Hz, H-7), 5.72 (1H, t, *J* = 5.4 Hz, H-6), 3.74 (6H, s, (−OCH_3_)_2_) 2.08 (3H, s, H-17), 2.03 (1H, d, *J* = 4.3 Hz, H-5), 1.99 (1H, td, *J* = 13.6, 4.6 Hz, H-1α), 1.61 (1H, qt, *J* = 13.4, 3.2 Hz, H-2ß), 1.52 (1H, dquint, *J* = 13.9, 4.0 Hz, H-2α), 1.38 (1H, dm, *J* = 12.8 Hz, H-3ß), 1.29 (1H, td, *J* = 12.8, 3.0 Hz, H-3α), 1.19 (3H, s, H-15), 1.17 (1H, dm, *J* = 12.8 Hz, H-1ß), 1.14 (3H, s, H-14), 1.00 (3H, s, H-13); ^13^C NMR (100 MHz, CDCl_3_) δ 174.6 (C, C-11), 170.5 (C, C-16), 167.1 (C, C-12), 136.9 (CH, C-7), 133.4 (C, C-8), 78.2 (C, C-9), 66.0 (CH, C-6), 52.8 (OCH_3_ of C-12), 52.5 (OCH_3_ of C-11), 44.4 (CH_2_, C-3), 44.3 (CH, C-5), 41.4 (C, C-10), 34.1 (C, C-4), 33.1 (CH_3_, C-13), 32.9 (CH_2_, C-1), 25.0 (CH_3_, C-14), 21.9 (CH_3_, C-17), 20.2 (CH_3_, C-15), 18.4 (C, C-2); HRESIMS *m/z* 391.17307 [M + Na]^+^ (calcd for C_19_H_28_O_7_Na, 391.17272).

**Cinnamo-N,11-dihydro-11-pyridazinol (7):** To a solution of **1** (50.0 mg, 0.162 mmol) in 0.5 mL CH_2_Cl_2_–EtOH (1:1) was added to a round-bottomed flask containing a solution of H_2_NNH_2_ • H_2_O (0.015 mL, 0.486 mmol) in 0.16 mL of EtOH at 0 °C via syringe pump. The reaction was warmed to room temperature and was allowed to proceed for 2 h. The reaction mixture was concentrated via rotary evaporation. Toluene (1 mL) was added to the crude material and the resulting mixture was stirred for 10 min at room temperature. The solvent was then removed by rotary evaporation and the process was repeated with CH_2_Cl_2_ (1 mL), yielding **7** as a yellow powder: ${[\alpha]}_{D}^{20}$ −303 (*c* 1, CH_3_CN); UV (CH_3_CN) λ_max_ (log ε) 286 (3.91), 249 (3.89) nm; IR (KBr) ν_max_ 3325, 2949, 2922, 2868, 1735, 1462, 1445, 1370, 1241, 1216, 1081, and 1029 cm^−1^; ^1^H NMR (400 MHz, CD_3_CN) δ 6.89 (1H, s, H-12), 6.06 (1H, s, 9-OH), 5.79 (1H, d, *J* = 5.0 Hz, H-7), 5.67 (1H, t, *J* = 4.8 Hz, H-6), 4.64 (1H, s, H-11), 3.63 (1H, br s, NH), 3.11 (1H, s, 11-OH), 2.13 (1H, d, *J* = 4.6 Hz, H-5), 2.028 (1H, td, *J* = 12.8, 4.1 Hz, H-1α), 2.025 (3H, s, H-17), 1.83 (1H, dq, *J* = 13.6, 2.8 Hz, H-1ß), 1.68 (1H, qt, *J* = 13.7, 3.3 Hz, H-2ß), 1.48 (1H, dquint *J* = 14.1, 3.5 Hz, H-2α), 1.37 (1H, dq, *J* = 12.8, 2.8 Hz, H-3ß), 1.274 (3H, s, H-15), 1.271 (1H, td, *J* = 13.1, 3.2 Hz, H-3α), 1.17 (3H, s, H-14), 0.99 (3H, s, H-13); ^13^C NMR (100 MHz, CD_3_CN) δ 171.7 (C=O, C-16), 140.9 (CH, C-12), 135.3 (C, C-8), 126.5 (CH, C-7), 82.2 (CH, C-11), 73.6 (C, C-9), 68.2 (CH, C-6), 46.5 (CH, C-5), 45.8 (CH_2_, C-3), 41.4 (C, C-10), 35.1 (C, C-4), 34.8 (CH_2_, C-1), 33.8 (CH_3_, C-13), 25.7 (CH_3_, C-14), 22.3 (CH_3_, C-17), 19.7 (C, C-2), 19.1 (CH_3_, C-15); HRESIMS *m/z* 323.19760 [M + H]^+^ (calcd for C_17_H_27_O_4_N_2_, 323.19653).

**7*ß*-hydroxy-cinnamopyridazine (18):** ^1^H-NMR analysis showed that compound **7** gradually converted to **18** upon incubation in deuterated MeOH at 0 ^o^C overnight (*ca.* 2.5:1 *ratio*, respectively). ^1^H NMR (400 MHz, MeOD) δ 9.24 (1H, s, H-11), 9.04 (1H, s, H-12), 5.82 (1H, br t, *J* = 1.5 Hz, H-6), 4.21 (1H, d, *J* = 1.5 Hz, H-7), 2.43 (1H, dq, *J* = 12.6, 1.9 Hz, H-1ß), 2.06 (3H, s, H-17), 2.01 (1H, dquint *J* = 10.2, 3.7 Hz, H-2β), 1.78 (1H, d, *J* = 0.56 Hz, H-5), 1.74 (1H, m, H-2α), 1.64 (3H, s, H-14), 1.60 (1H, dm, *J* = 13.6 Hz, H-3ß), 1.43 (1H, td, *J* = 12.5, 3.9 Hz, H-1α), 1.38 (1H, td, *J* = 14.0, 3.8 Hz, H-3α), 1.15 (3H, s, H-15), 1.11 (3H, s, H-13); ^13^C NMR (100 MHz, MeOD) δ 172.7 (C=O, C-16), 155.4 (CH, C-12), 151.6 (CH, C-11), 150.7 (C, C-9), 134.3 (C, C-8), 77.7 (CH, C-7), 69.3 (CH, C-6), 49.0 (CH, C-5), 44.4 (CH_2_, C-3), 41.5 (CH_2_, C-1), 38.6 (C, C-10), 35.5 (C, C-4), 34.2 (CH_3_, C-13), 27.0 (CH_3_, C-14), 24.4 (CH_3_, C-15), 22.1 (CH_3_, C-17), 20.7 (C, C-2).

**Cinnamodial 12-ethylene acetal (11):** To a 10 mL round-bottom flask containing 4 Å molecular sieves, cinnamodial (50 mg, 0.162 mmol) was added and dissolved in anhydrous benzene (6 mL). Ethylene glycol (9.04 µL, 0.162 mmol) and a crystal of *p*-toluenesulfonic acid (1 mg) were added and the solution refluxed for 12 h. The reaction mixture was cooled, ethyl acetate (6 mL) was added and the solution was washed with saturated NaHCO_3_, dried over magnesium sulfate, and evaporated. The residue was purified via column chromatography using a gradient of hexanes–EtOAc (from 4:1 to 2:1) and preparative TLC developed with hexanes–EtOAc (2:1) to afford acetal **11** (3.9 mg, 7%) as fine colorless crystals: ${[\alpha]}_{D}^{20}$ −157 (*c* 1, ACN); IR (KBr) ν_max_ 3485, 2950, 2923, 2871, 1733, 1463, 1390, 1370, 1241, 1213, 1126, 1086, 1062, 1023, 977, 951, 919, and 802 cm^−1^; ^1^H NMR (400 MHz, CDCl_3_) δ 9.87 (1H, d, *J* = 0.72 Hz, H-11), 6.27 (1H, d, *J* = 4.9 Hz, H-7), 5.68 (1H, t, *J* = 4.7 Hz, H-6), 5.17 (1H, s, H-12), 3.90 (2H, m, 12-OCH_2_CH_2_O-), 3.89 (1H, d, *J* = 0.84 Hz, 9-OH), 3.81 (2H, m, 12-OCH_2_CH_2_O-), 2.08 (3H, s, H-17), 2.05 (1H, d, *J* = 4.6 Hz, H-5), 1.87 (1H, td, *J* = 13.3, 4.4 Hz, H-1α), 1.62 (1H, qt, *J* = 13.5, 3.3 Hz, H-2ß), 1.48 (1H, dquint, *J* = 13.8, 3.7 Hz, H-2α), 1.47 (3H, s, H-15), 1.36 (1H, dq, *J* = 13.2, 1.4 Hz, H-3ß), 1.27 (1H, td, *J* = 13.6, 2.9 Hz, H-3α), 1.15 (3H, s, H-14), 0.99 (3H, s, H-13), 0.97 (1H, dm, *J* = 12.8 Hz, H-1ß); ^13^C NMR (100 MHz, CDCl_3_) δ 203.0 (CH, C-11), 170.5 (C, C-16), 136.7 (C, C-8), 131.7 (CH, C-7), 105.5 (CH, C-12), 80.3 (C, C-9), 66.6 (CH, C-6), 65.4 (CH_2_, 12-OCH_2_CH_2_O-), 65.0 (CH_2_, 12-OCH_2_CH_2_O-), 44.9 (CH, C-5), 44.4 (CH_2_, C-3), 41.9 (C, C-10), 34.2 (C, C-4), 32.9 (CH_3_, C-13), 32.9 (CH_2_, C-1), 25.0 (CH_3_, C-14), 22.0 (CH_3_, C-17), 20.4 (CH_3_, C-15), 18.2 (C, C-2); HRESIMS *m/z* 375.17801 [M + Na]^+^ (calcd for C_19_H_28_O_6_Na, 375.17781). Data agree with literature [1].

**Attempted base-induced ring opening of compound** **8 to compound 10**: DBU (127.23 µL, 0.851 mmol, MW 152.24, 1.018 g/mL) was added to a stirred solution of compound **8** (13.8 mg, 0.0425, MW 324.42) in dry toluene (1 mL) at room temperature under argon. The mixture was heated at reflux for 2.5 h. Reaction monitoring by TLC showed no change in the starting material. After 2.5 h the reaction was cooled and the mixture was directly subjected to column chromatography eluting with EtOAc in order to remove excess DBU. The fractions were pooled and concentrated *in vacuo* to yield the starting material, as observed by TLC and NMR.

# Cinnamodial (1),

Characterization of (**1**) – Cinnamodial (**1**) All data match published literature[2,3].

**CDCl_3_**

**CDCl_3_**

**Figure S5**. Proton NMR spectra (top) and carbon NMR spectra (bottom) of compound **1** in CDCl_3_ at 400 and 100 MHz, calibrated to Chloroform-d 7.26 (1) and 77.36 (3).

# Cinnamodiacid (5),

Followed the Pinnick oxidation protocol which was used by Fotsop *et al*. **2008** to oxidize isodrimanial to the diacid derivative[4].

**MeOD**

**Water**

**MeOD**

**Water**

**Figure S6**. Proton NMR spectra (Full and inset) of compound **5** in CD_3_OD at 400 MHz, calibrated to Methanol-d_4_ 3.34 (5).

**MeOD**

**Figure S7**. Carbon NMR spectra of compound **5** in CD_3_OD at 100 MHz, calibrated to Methanol-d_4_ 49.86 (7).


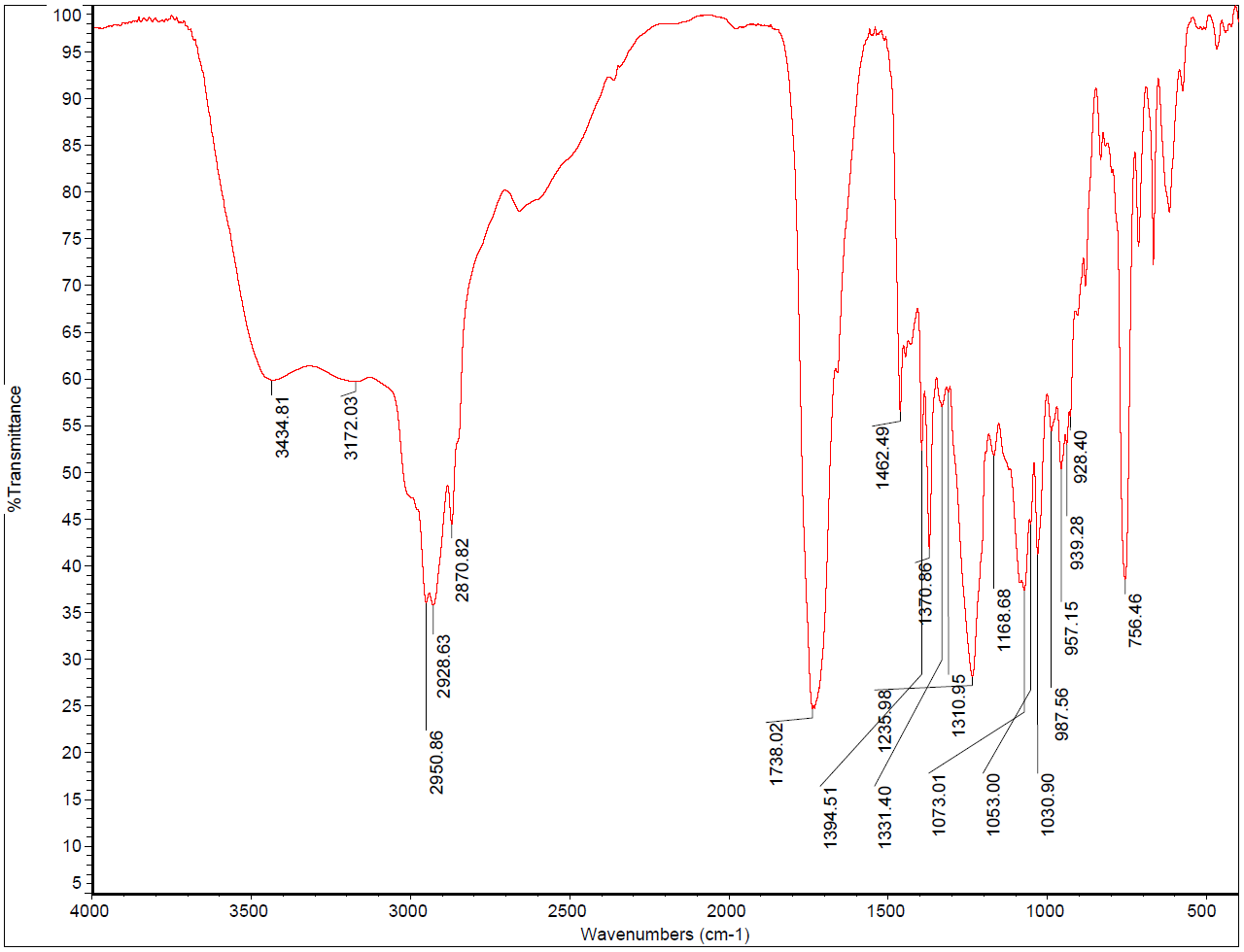


**Figure S8**. IR spectrum of compound **5**.

**Figure S9**. HRESIMS of compound **5**.

# Cinnamodimethylester (6),

Followed the esterification protocol which was used by Matsuda *et al*. **2002** to esterify the diacid of polygodial to the dimethylester derivative[5].

**CDCl_3_**

**CDCl_3_**

**Figure S10**. Proton NMR spectra (Full and inset) of compound **6** in CDCl_3_ at 400 MHz, calibrated to Chloroform-d 7.26 (1).

**CDCl_3_**

**Figure S11**. Carbon NMR spectra of compound **6** in CDCl_3_ at 100 MHz, calibrated to Chloroform-d 77.36 (3).


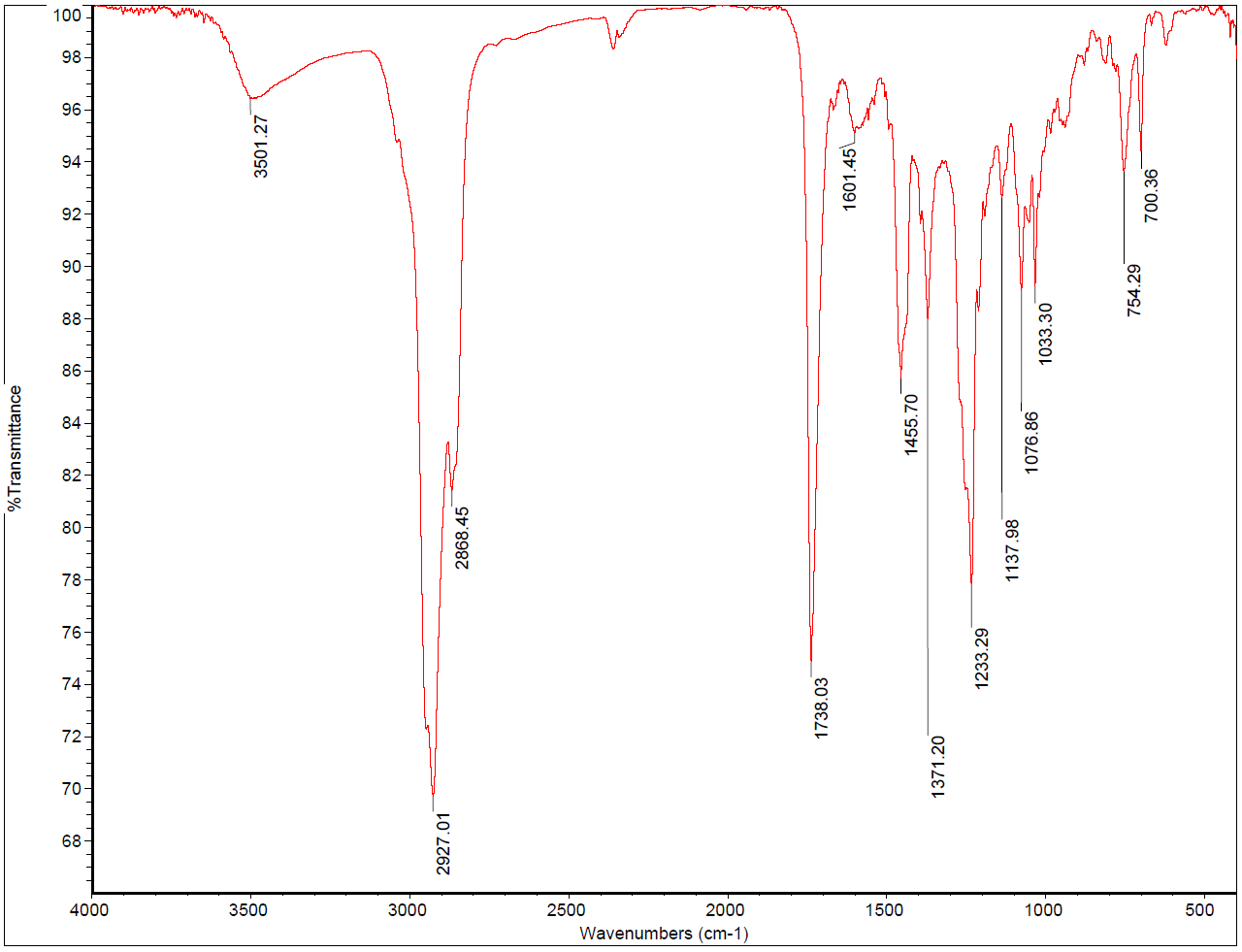


**Figure S12**. IR spectrum of compound **6**.

**Figure S13**. HRESIMS of compound **6**.

# Cinnamo-N,11-dihydro-11-pyridazinol (7),

Followed the 1,2-diazine protocol which was used by Montavon *et al*. **2013** to prepare benzo[g]phthalazine from naphthalene-2,3-dicarbaldehyde[6].

**Figure S14**. Proton NMR spectra of compound **7** in CD_3_CN (top), calibrated to acetonitrile-d_3_ 1.96 (5), and CD_3_OD (bottom), calibrated to Methanol-d_4_ 3.34 (5), at 400 MHz.

**Figure S15**. Carbon NMR spectra of compound **7** in CD_3_CN at 100 MHz, calibrated to acetonitrile-d_3_ 118.77 (1).


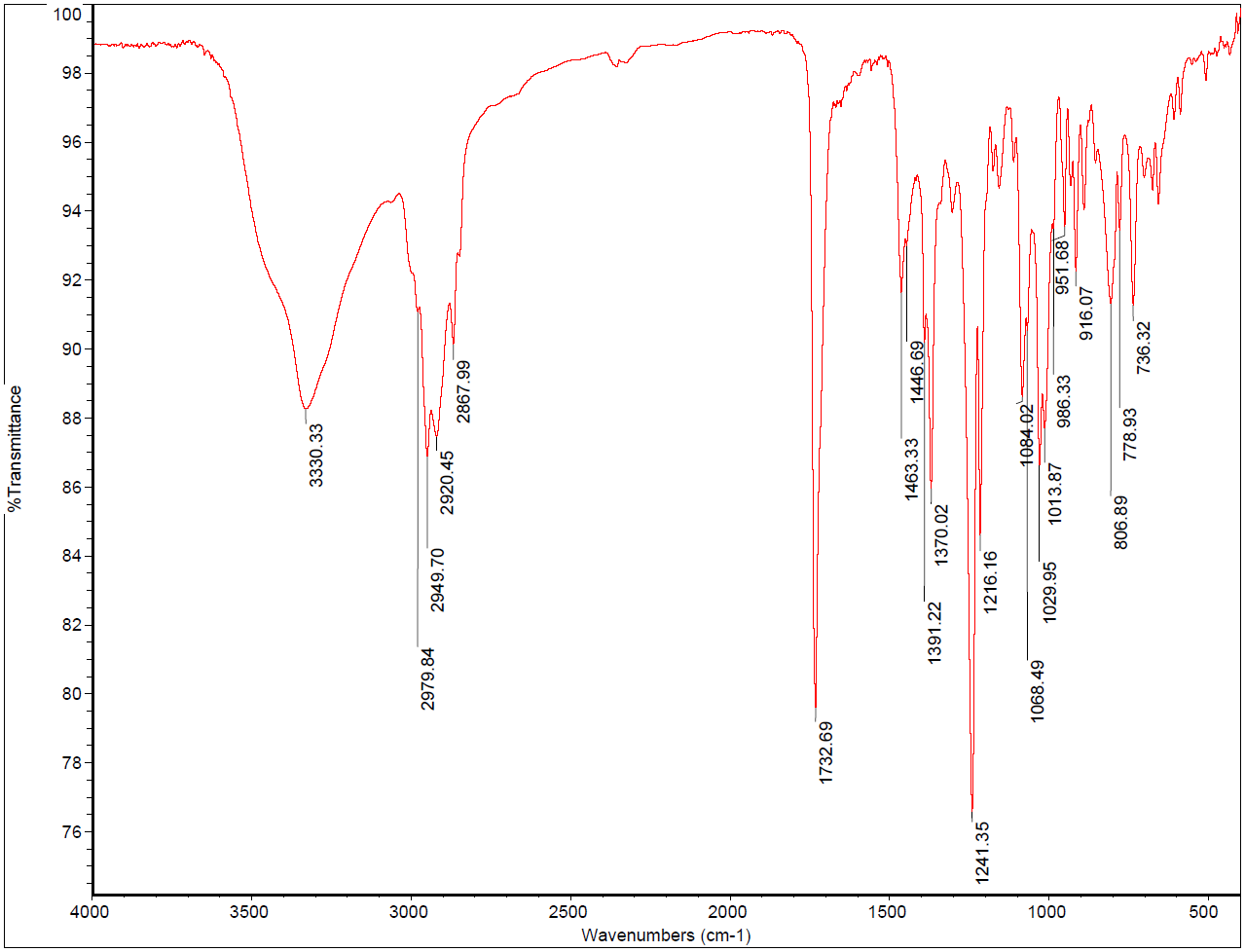


**Figure S16**. IR spectrum of compound **7**.

**Figure S17**. HRESIMS of compound **7**.

# 7β-hydroxy-cinnamopyridazine (18),

**Figure S18**. Proton NMR spectra (top) and carbon NMR spectra (bottom) of compound **18** in CD_3_OD at 400 and 100 MHz, calibrated to calibrated to Methanol-d_4_ 3.34 (5) and 49.86 (7), respectively.


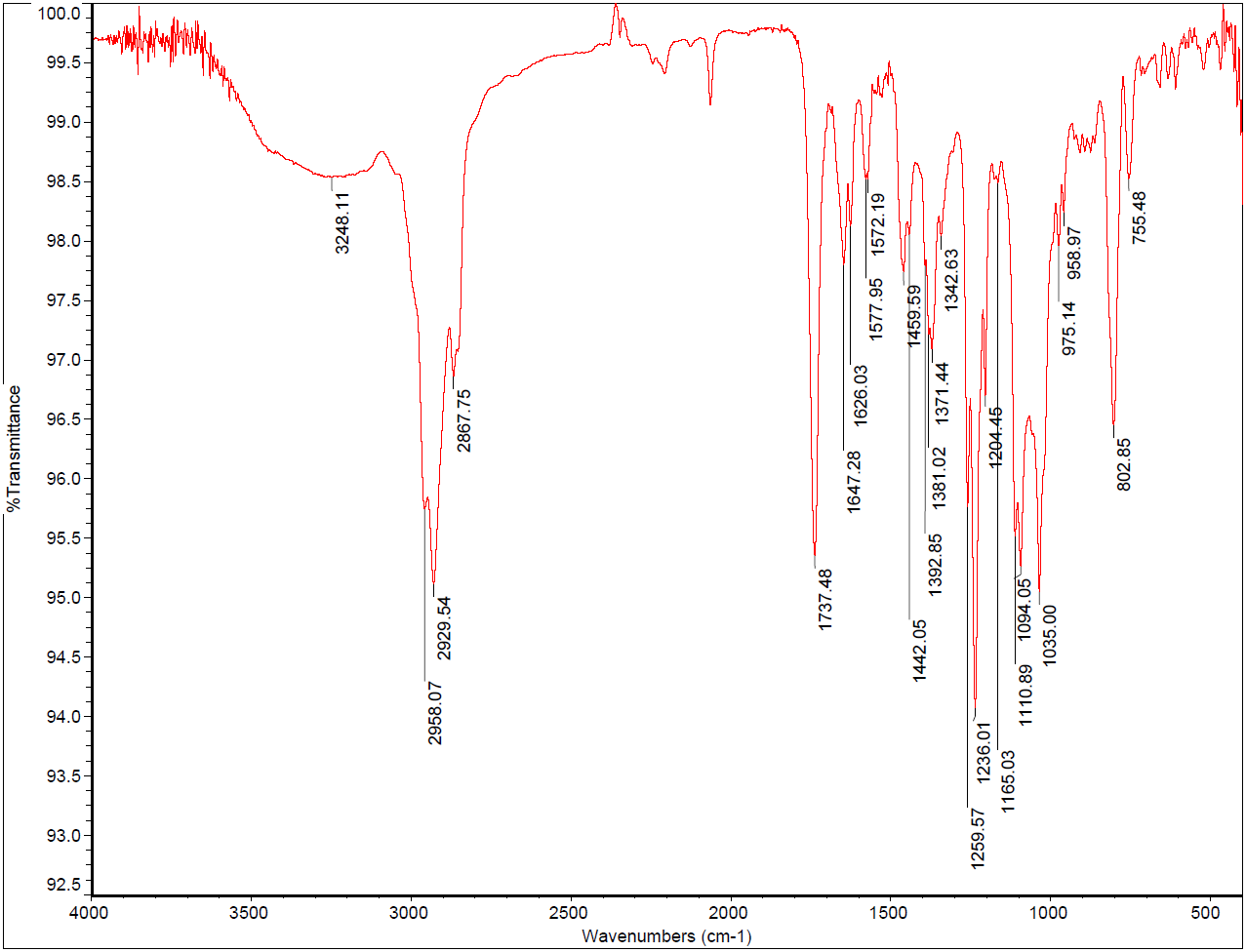


**Figure S19**. IR spectrum of compound **18**.

******

# 6-O-acetyl-12α-methyl-pereniporin A (8),

**CDCl_3_**

**Figure S20**. Proton NMR spectra (Full and inset) of compound **8** in CDCl_3_ at 400 MHz, calibrated to Chloroform-d 7.26 (1).

**CDCl_3_**

**Figure S21**. Carbon NMR spectra of compound **8** in CDCl_3_ at 100 MHz, calibrated to Chloroform-d 77.36 (3).

**CDCl_3_**

**Figure S22**. Proton NMR spectra (Full and inset) of compound **8** in CDCl_3_ at 400 MHz, calibrated to Chloroform-d 7.26 (1). Proton taken after 46 day incubation in deuterated chloroform.


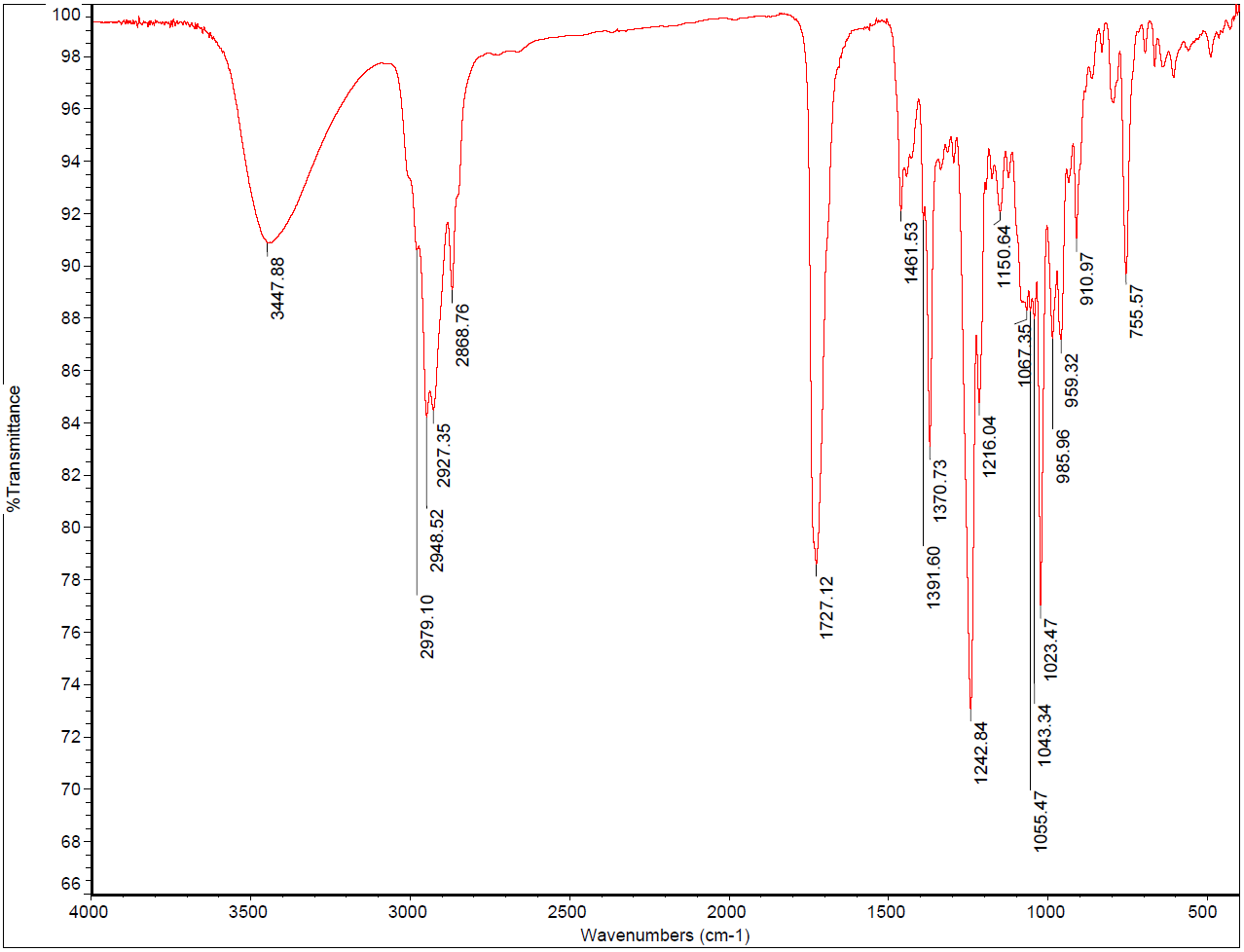


**Figure S23**. IR spectrum of compound **8**.

******

**Figure S24**. HRESIMS of compound **8**.

# 6-O-acetyl-12a-methyl-pereniporin A (9),

**CDCl_3_**

**Figure S25**. Proton NMR spectra (Full and inset) of compound **9** in CDCl_3_ at 400 MHz, calibrated to Chloroform-d 7.26 (1).

**CDCl_3_**

**Figure S26**. Carbon NMR spectra of compound **9** in CDCl_3_ at 100 MHz, calibrated to Chloroform-d 77.36 (3).


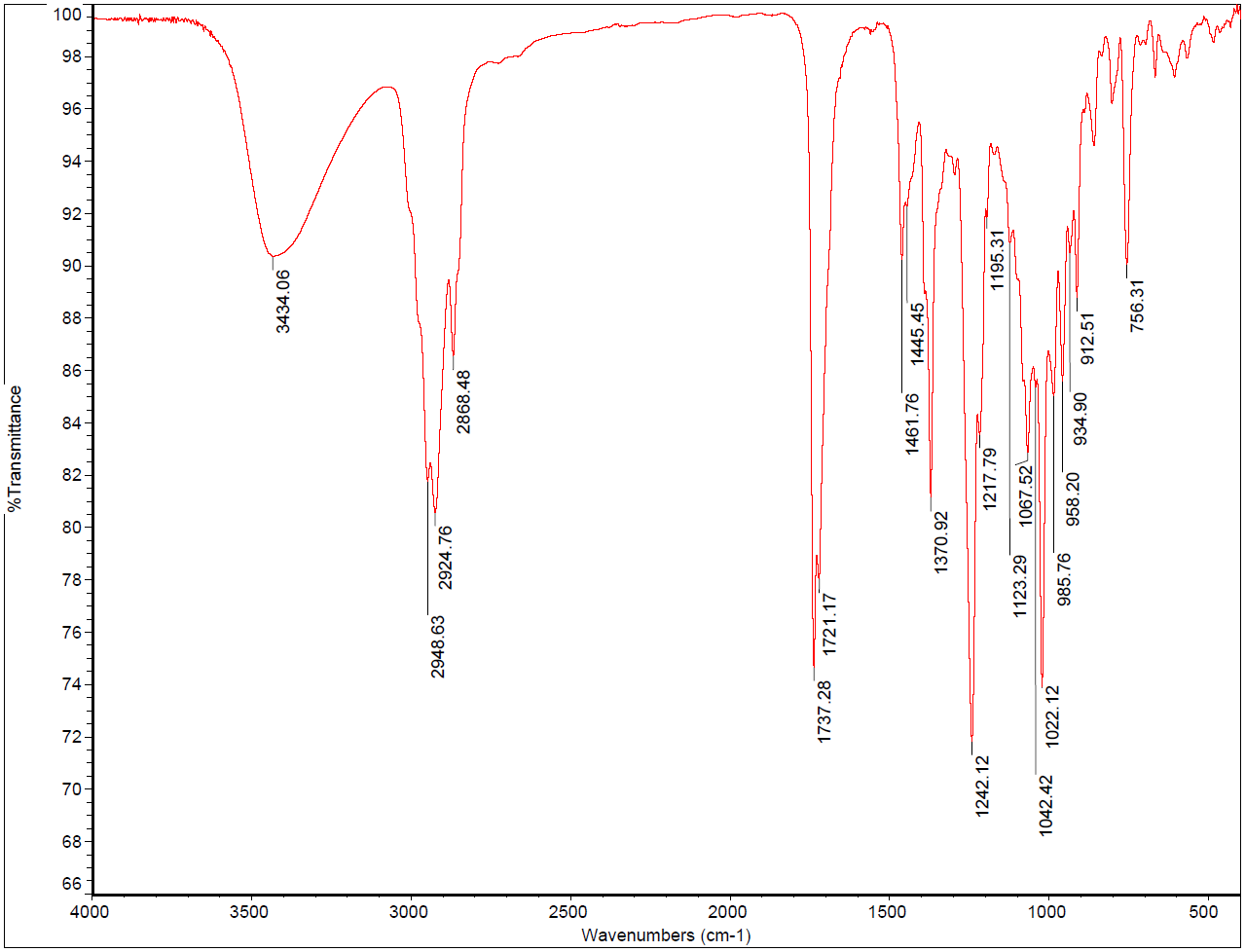


**Figure S27**. IR spectrum of compound **9**.

**Figure S28**. HRESIMS of compound **9**.

(-)-6ß-Acetoxy-9α-hydroxydrim-7-ene-12-methyl-12-one-11-al (10), ****

**CDCl_3_**

**CDCl_3_**

**Figure S29**. Proton NMR spectra (top) and carbon NMR spectra (bottom) of compound **10** in CDCl_3_ at 400 and 100 MHz, calibrated to Chloroform-d 7.26 (1)and 77.36 (3), respectively.


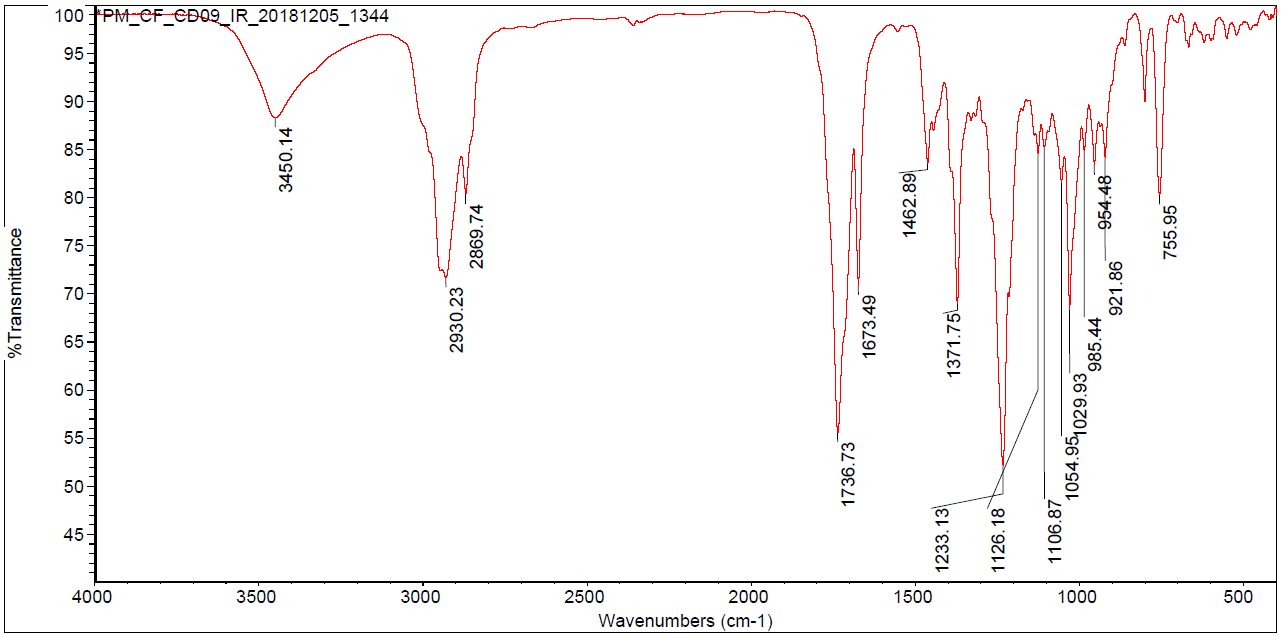


**Figure S30**. IR spectrum of compound **10**.


**Figure S31**. HRESIMS of compound **10**.

# Cinnamodial 12-ethylene acetal (11),

Followed the protocol which was used by Garlaschelli *et al*. **1991**[1].

******Figure S32***.* Proton NMR spectra (top) and carbon NMR spectra (bottom) of compound **11** in CDCl_3_ at 400 MHz and 100 MHz, calibrated to Chloroform-d 7.26 (1)and 77.36 (3), respectively.

**Ethyl acetate**

**CDCl_3_**

**Ethyl acetate**

**Ethyl acetate**

**Ethyl acetate**

**CDCl_3_**


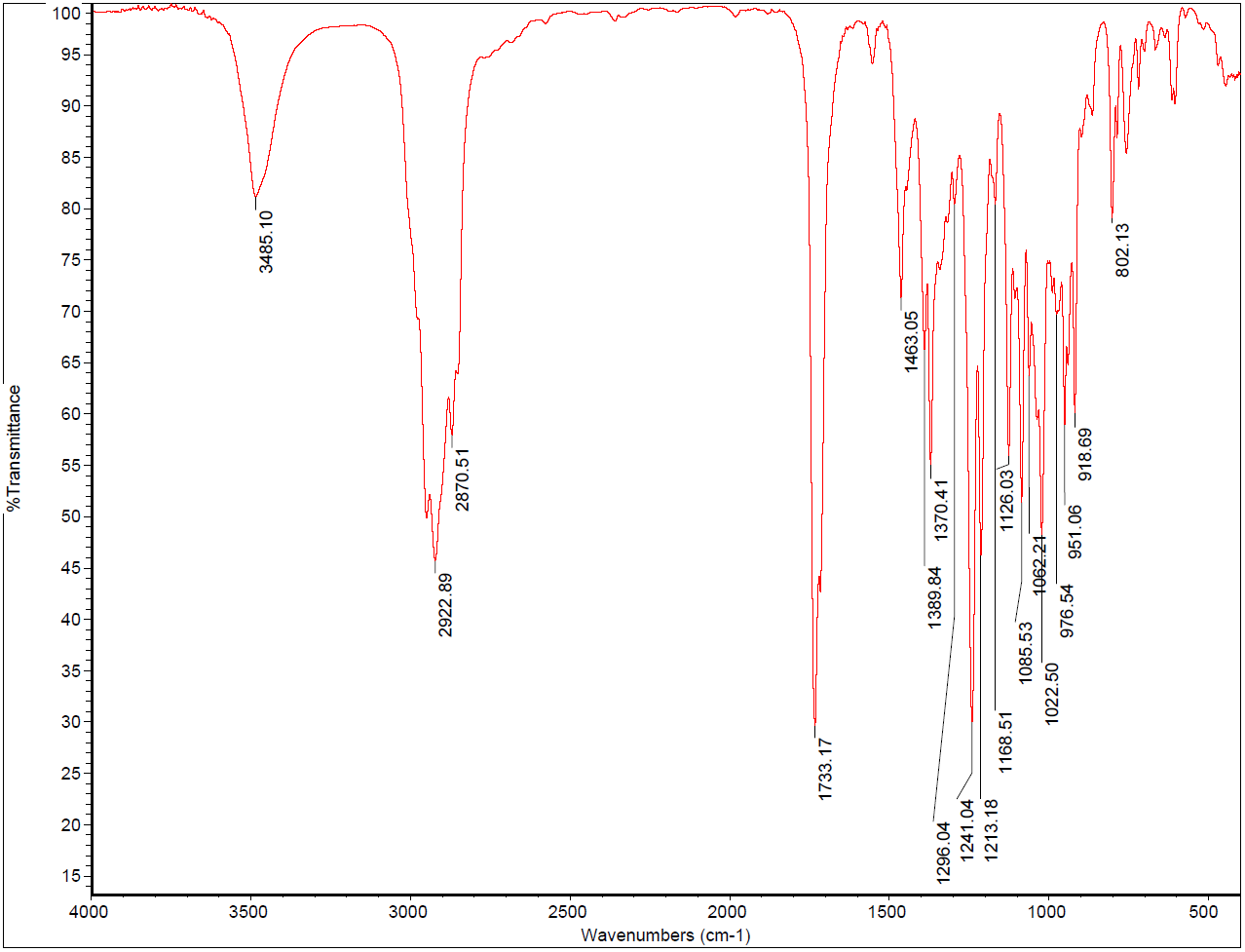


**Figure S33**. IR spectrum of compound **11**.

******

**Figure S34**. HRESIMS of compound **11**.

# 12α/ß-methyl-pereniporin A (12),

**Figure S35**. NMR spectra (Full and inset) of compound **12** in CDCl_3_ at 400 MHz, calibrated to Chloroform-d 7.26 (1).

**Figure S36**. Carbon NMR spectra (full and inset) of compound **12** in CDCl_3_ at 100 MHz, calibrated to Chloroform-d 77.36 (3).


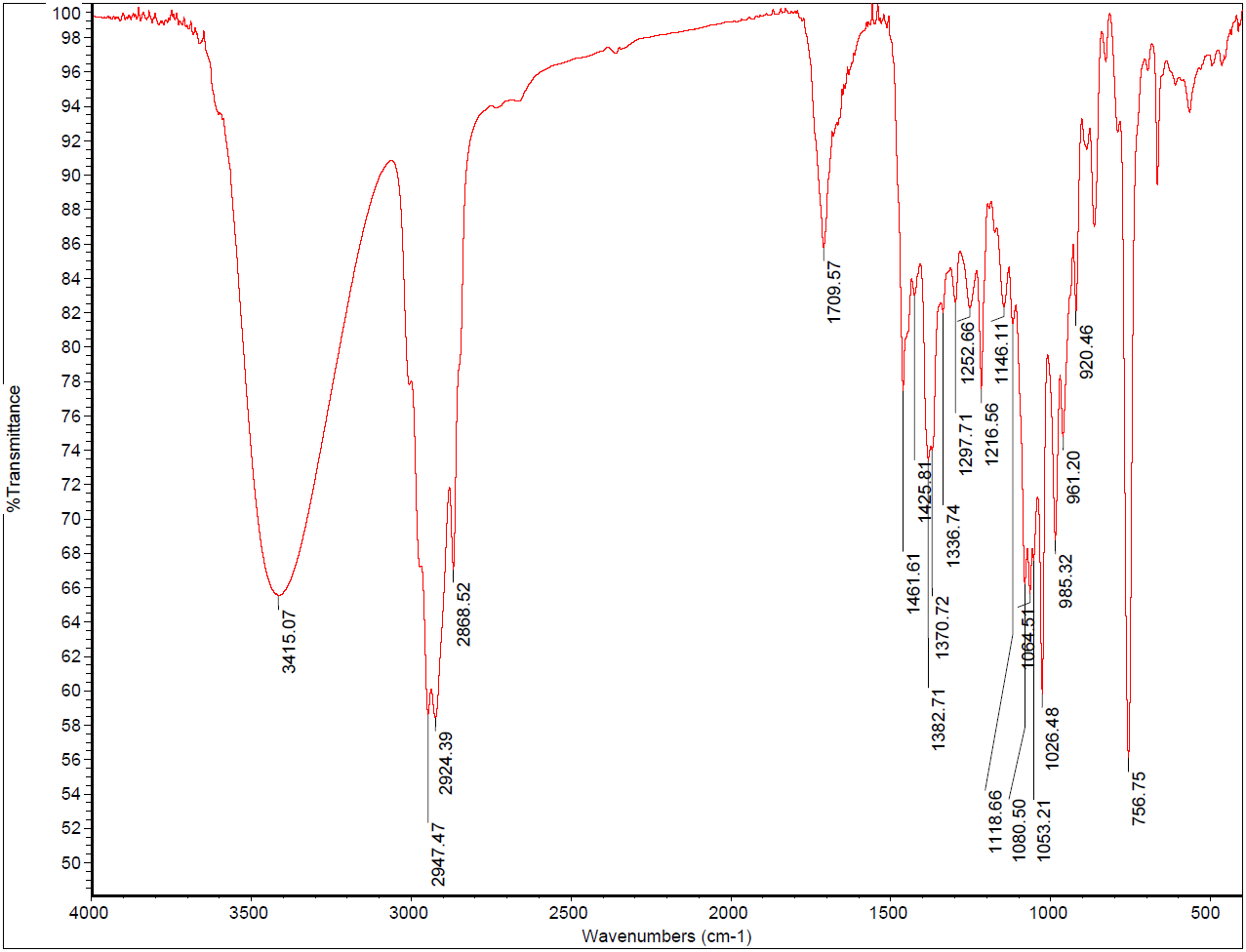


**Figure S37**. IR spectrum of compound **12**.

**Figure S38**. HRESIMS of compound **12**.

(1’S/R)-1’-((8aS)-5,5,8a-trimethyl-1,4-dioxo-1,4,4a,5,6,7,8,8a-octahydronaphthalene-2-yl)ethyl formate (13),


**Figure S39**. NMR spectra (Full and inset) of compound **13** in CDCl_3_ at 400 MHz, calibrated to Chloroform-d 7.26 (1).

**Figure S40**. Carbon NMR spectra (full and inset) of compound **13** in CDCl_3_ at 100 MHz, calibrated to Chloroform-d 77.36 (3).


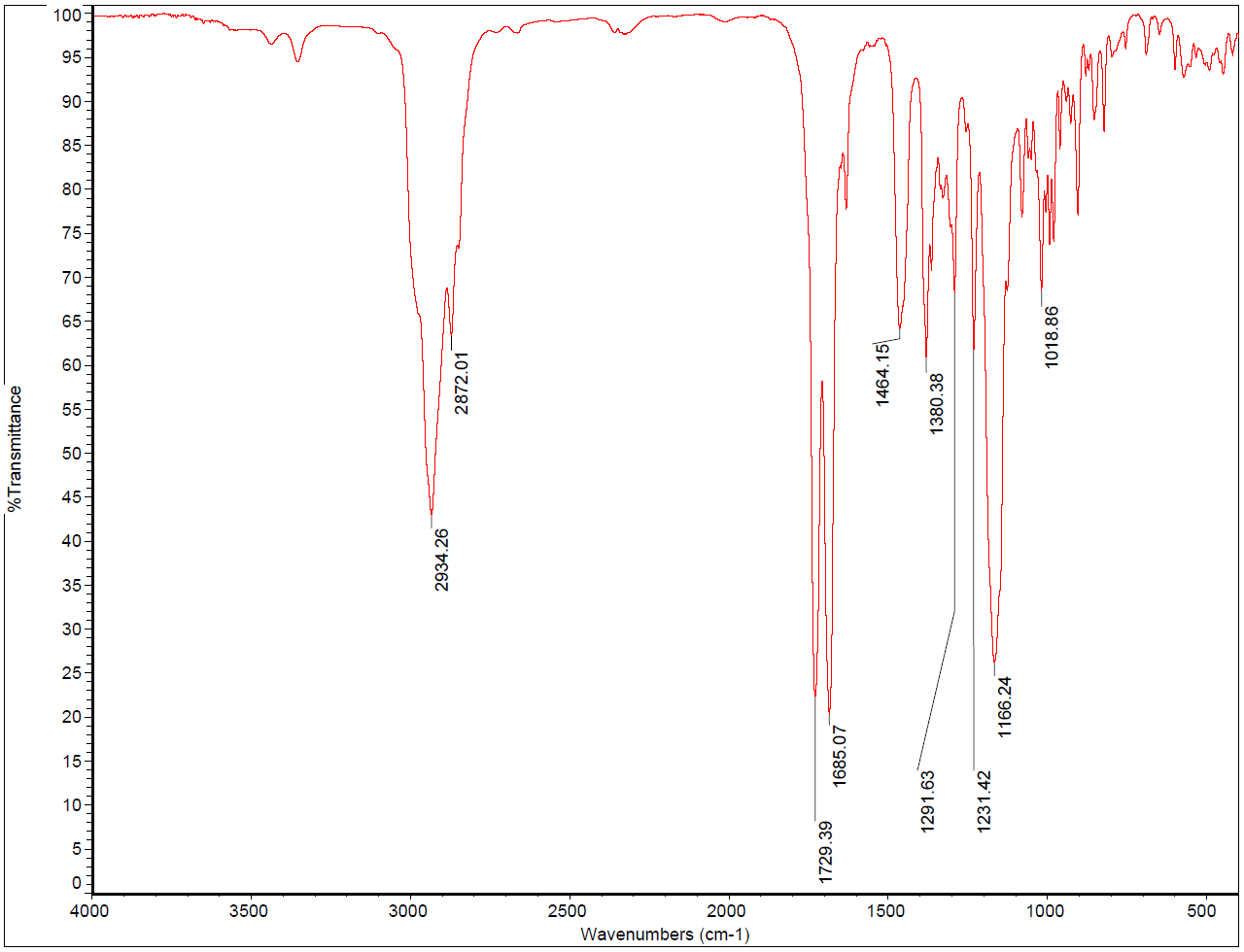


**Figure S41**. IR spectrum of compound **13**.

**Figure S42**. HRESIMS of compound **13**.

# Cinnamothiazolidine (16),

Compare to the reaction product of muzigadial with *L*-cysteine methyl ester done by Lam and Frazier[7].

**Figure S43**. Proton NMR spectra (top) and carbon NMR spectra (bottom) of compound **16** in Pyridine at 400 MHz and 100 MHz, calibrated to Pyridine-d_5_ 7.22 (1) and 123.87 (3), respectively.

******
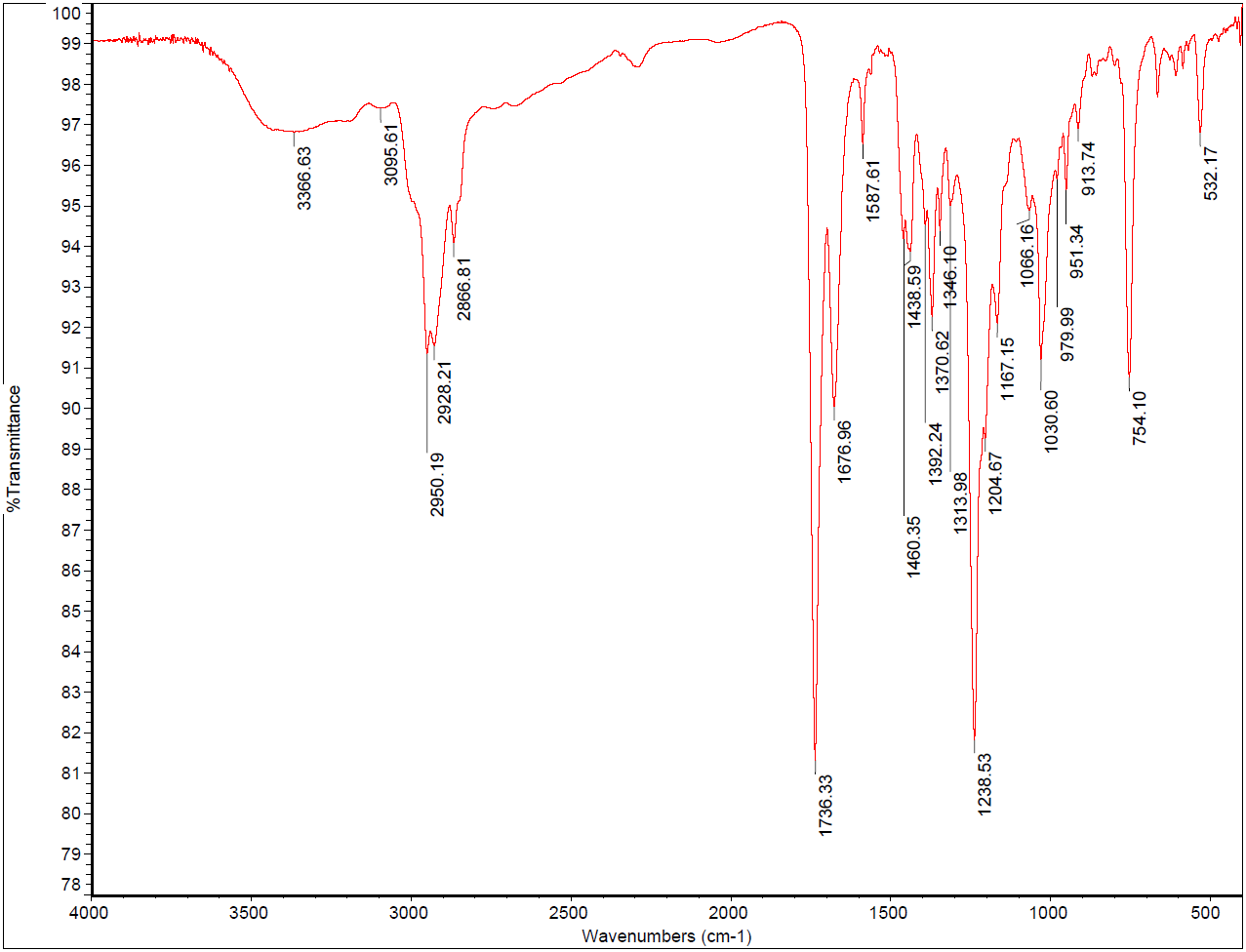


**Figure S44** IR spectrum of compound **16**.

**Figure S45**. HRESIMS of compound **16**.

# References

1. Garlaschelli L, Tullio P de, Vidari G. Synthetic studies on biologically active natural compounds. Part III. Stereospecific transformation of uvidin A into (−)-cinnamosmolide. Tetrahedron. 1991;47: 6769–6776. doi:10.1016/S0040-4020(01)82328-0

2. Canonica L, Corbella A, Jommi G, Křepinský J, Ferrari G, Casagrande C. The structure of cinnamolide, cinnamosmolide and cinnamodial, sesquiterpenes with drimane skeleton from *Cinnamosma fragrans* Baillon. Tetrahedron Lett. 1967;8: 2137–2141. doi:10.1016/S0040-4039(00)90783-4

3. Canonica L, Corbella A, Gariboldi P, Jommi G, Krˇepinský J, Ferrari G, et al. Sesquiterpenoids of *Cinnamosma fragrans* Baillon: Structure of cinnamolide, cinnamosmolide and cinnamodial. Tetrahedron. 1969;25: 3895–3902. doi:10.1016/S0040-4020(01)82921-5

4. Fotsop DF, Roussi F, Le Callonec C, Bousserouel H, Litaudon M, Guéritte F. Isolation and characterization of two new drimanes from *Zygogynum baillonii* and synthesis of analogues. Tetrahedron. 2008;64: 2192–2197. doi:10.1016/j.tet.2007.12.022

5. Matsuda H, Pongpiriyadacha Y, Morikawa T, Kashima Y, Nakano K, Yoshikawa M. Protective effects of polygodial and related compounds on ethanol-induced gastric mucosal lesions in rats: Structural requirements and mode of action. Bioorg Med Chem Lett. 2002;12: 477–482.

6. Montavon TJ, Türkmen YE, Shamsi NA, Miller C, Sumaria CS, Rawal VH, et al. [2+2+2] Cycloadditions of Siloxy Alkynes with 1,2-Diazines: From Reaction Discovery to Identification of an Antiglycolytic Chemotype. Angew Chem Int Ed. 2013;52: 13576–13579. doi:10.1002/anie.201305711

7. Lam PY-S, Frazier JL. Model study on the mode of action of muzigadial antifeedant. Tetrahedron Lett. 1987;28: 5477–5480. doi:10.1016/S0040-4039(00)96758-3
